# Time-restricted eating and metabolic health: implications for nutritional strategies and weight loss

**DOI:** 10.3389/fnut.2026.1872454

**Published:** 2026-06-11

**Authors:** Bianca Camilo Schimenes, Tathiana A. Alvarenga, Sergio Brasil Tufik, Sergio Tufik, Monica Levy Andersen

**Affiliations:** 1 Departamento de Psicobiologia, Universidade Federal de São Paulo (UNIFESP), São Paulo, Brazil; 2 Instituto do Sono, Associação Fundo de Incentivo à Pesquisa (AFIP), São Paulo, Brazil

**Keywords:** sleep, cardiometabolic health, chrononutrition, chronotype, dietary intervention, nutrition, time-restricted eating

## Abstract

Time-restricted eating (TRE) has emerged as a promising dietary strategy within the field of chrononutrition, focusing on the temporal organization of food intake rather than caloric restriction alone. This mini review summarizes current evidence on the effects of TRE on body weight, metabolic health, and circadian regulation. Experimental and clinical studies suggest that TRE, typically involving daily eating windows of 6–10 h, may promote modest weight loss and improvements in glycemic control, insulin sensitivity, and lipid profile. Some effects have been observed even in the absence of significant weight loss, supporting a potential role of meal timing in cardiometabolic regulation, although findings are still inconsistent. Data indicates that many of the identified advantages appear to be largely driven by spontaneous caloric restriction, with several randomized controlled trials showing no additional benefits of TRE compared to conventional hypocaloric diets. The timing and duration of the eating window might influence outcomes, with early TRE and moderate eating windows having more favorable results than delayed or extreme protocols. Circadian biology provides a mechanistic framework for understanding these effects, as alignment between feeding-fasting cycles and endogenous rhythms can enhance metabolic efficiency. Most studies do not account for individual chronotype, and evidence on circadian, inflammatory, and behavioral outcomes remains limited. TRE represents a feasible and non-invasive dietary approach with potential health benefits, particularly when compared to unrestricted eating patterns, although its superiority over caloric restriction alone has not been fully elucidated. Future research should prioritize long-term interventions, standardized protocols, and the integration of circadian factors, particularly chronotype, to better elucidate the role of TRE in metabolic health and clinical practice.

## Graphical abstract

**Figure:**
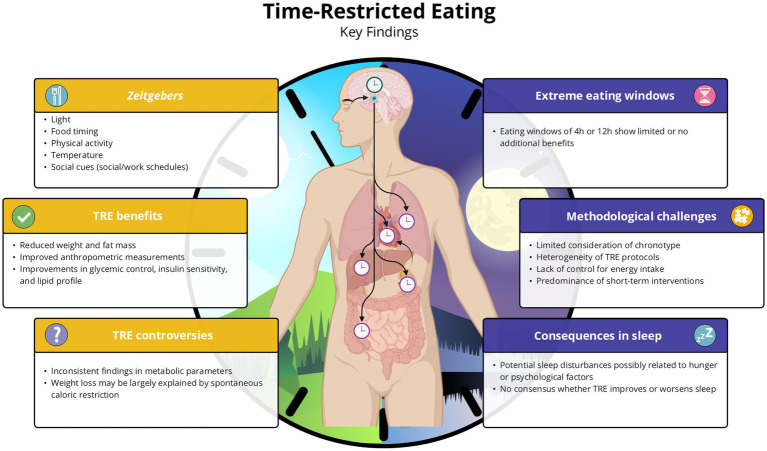
This graphical abstract summarizes current evidence on time-restricted eating (TRE). TRE, defined as limiting daily food intake to a consistent eating window, is associated with modest weight loss and improvements in cardiometabolic and physiological outcomes. These effects may be partly explained by spontaneous caloric restriction and alignment with circadian rhythms. However, findings remain heterogeneous, particularly when compared with caloric restriction, suggesting a primary role of energy deficit. Variability in study design and intervention protocols contributes to inconsistent results, highlighting the need for further research. Figure created with Biorender, Canva, and Freepik.

## Introduction

Obesity is widely recognized as a chronic, multifactorial disease characterized by excess adiposity and a persistent state of low-grade systemic inflammation (1). Over the past decades, its prevalence has increased dramatically, reaching epidemic proportions worldwide. Recent global estimates revealed that approximately 43% of adults are overweight and 16% are living with obesity, corresponding to more than 2.5 billion overweight adults, including nearly 890 million with obesity (2). This condition frequently coexists with cardiometabolic disorders, such as type 2 diabetes mellitus (T2DM), hypertension, and cardiovascular disease, and is associated with an elevated risk of premature mortality (3, 4).

The development of obesity is driven by a complex interaction of behavioral, environmental, and biological factors. Positive energy balance caused by excessive caloric intake, reduced physical activity, high consumption of ultra-processed foods, and genetic susceptibility are well-established contributors (5–7). Growing evidence indicates that not only the quantity and quality of food intake, but also the timing of eating may influence body weight regulation and metabolic health (8, 9). Recent studies found that misalignment between food intake and endogenous circadian rhythms can impair metabolic regulation, potentially increasing the risk of weight gain and metabolic disorders (10, 11).

Circadian rhythms regulate daily oscillations in several physiological processes, such as metabolism, hormone secretion, and energy utilization (12). One behavioral manifestation of circadian organization is chronotype, defined as an individual’s preferred timing for sleep and daily activities (13). Chronotype reflects interindividual differences in circadian phase, with some individuals exhibiting earlier (“morning”) patterns and others showing delayed (“evening”) rhythms (14). These variations reflect differences in the temporal organization of physiological and behavioral processes across individuals, including the timing of daily activities, such as sleep and food intake.

Alternative strategies to support weight management and metabolic health are increasingly being investigated. While conventional approaches primarily emphasize caloric restriction and intensified physical activity, growing attention has been directed toward dietary patterns that modify the temporal organization of food intake (8–10, 15, 16).

Time-restricted eating (TRE) has emerged as one such strategy. TRE involves limiting daily food intake to a consistent eating window, thereby extending the overnight fasting period (17). Importantly, some benefits were documented even in the absence of significant weight loss, suggesting that the timing of food intake may play an independent role in metabolic regulation (18). These potential improvements can result partly from interactions between feeding-fasting cycles and circadian rhythms (19). Aligning food intake with endogenous circadian patterns could therefore contribute to improved metabolic efficiency and reduced cardiometabolic risk. As a result, TRE has gained considerable attention over the past decade as a promising, non-invasive dietary approach for obesity management and health promotion.

This mini review summarizes the current body of data on TRE and metabolic health, highlighting its potential implications for nutritional strategies and weight loss. Unlike previous reviews primarily centered on metabolic outcomes, this mini review emphasizes the translational and clinical implications of TRE by integrating circadian biology, chronotype, sleep, and behavioral aspects relevant to personalized nutritional strategies.

## Circadian rhythms and chrononutrition

The circadian system coordinates daily rhythms in physiology and metabolism through a central clock located in the suprachiasmatic nucleus (SCN) of the hypothalamus and peripheral clocks distributed across metabolic tissues (20). These biological clocks regulate daily oscillations in several metabolic processes, including glucose and lipid metabolism, and hormone secretion. Their timing is influenced by external cues, known as zeitgebers, which synchronize internal circadian rhythms with environmental signals, such as light exposure, temperature cycles, and food intake (20).

Across the 24-h cycle, multiple physiological processes follow predictable circadian patterns. During the biological morning and daytime, insulin sensitivity, glucose tolerance, and diet-induced thermogenesis tend to be higher, favoring more efficient nutrient utilization (21–24). In contrast, during the evening and biological night, reductions in insulin sensitivity and metabolic rate are commonly observed, while melatonin secretion increases, promoting fasting and energy conservation (25–29). These coordinated oscillations illustrate how the circadian system organizes daily metabolic physiology and help explain why the timing of food intake appears to influence health outcomes.

Circadian disruption is increasingly common in modern societies. It is characterized by a misalignment between behavior and internal circadian timing or external environmental cues, which can lead to desynchronization among the central and peripheral clocks across different tissues (30). This disruption likely arises from extended working hours, shift work, irregular sleep–wake schedules, nighttime light exposure, and late meal timing (31–33). Misalignment of circadian rhythms has been linked with a wide range of adverse health outcomes, such as sleep–wake disorders, psychiatric and neurological conditions, metabolic and cardiovascular diseases, immune dysfunction, cancer, and gastrointestinal disorders (30, 33, 34).

Recent studies have drawn attention to the importance of chrononutrition in energy balance and metabolic regulation. A review examining the interaction between circadian rhythms and eating patterns pointed out that the timing of food intake relative to endogenous circadian rhythms can influence metabolic pathways involved in glucose regulation, lipid metabolism, and energy expenditure (8). Desynchrony between feeding-fasting cycles and the circadian system may contribute to metabolic dysregulation and weight gain, supporting the potential role of chrononutrition strategies, including TRE, in cardiometabolic health and body weight regulation (8).

Research on dietary strategies aligned with an individual’s chronotype is still emerging. Dinu et al. (15) proposed a study protocol to investigate the effects of a chronotype-adjusted diet on weight loss, cardiometabolic health, and gut microbiota, compared with a conventional calorie-restricted diet. In line with this approach, Muñoz et al. (35) demonstrated that aligning dietary timing with chronotype resulted in greater reductions in body weight, body mass index (BMI), and waist circumference than a conventional hypocaloric diet in individuals with overweight or obesity.

Recently published papers have explored the relationship between chronotype and weight-related outcomes during weight-loss interventions. For instance, a study evaluating participants enrolled in a behavioral weight management program found that individuals lost an average of approximately 6.3% of their baseline body weight over 16 weeks. Although evening chronotype was connected with slightly lower adherence to physical activity goals, chronotype itself was not significantly associated with weight loss or adherence to caloric intake targets, suggesting that weight-loss outcomes in structured behavioral approaches could occur independently of chronotype (36).

Taken together, these findings propose that circadian biology and individual temporal preferences may influence metabolic responses to dietary interventions. Novel weight-management strategies that consider the individual’s circadian rhythm and timing of food intake are being widely investigated.

## What is time-restricted eating? Concepts and protocols

TRE is a dietary strategy that limits daily food intake to a consistent time window, typically ranging from 6–11 h and lasting for 4–12 weeks, thereby extending the overnight fasting period. This approach is designed to align feeding-fasting cycles with the circadian system, potentially supporting metabolic regulation and overall health, independently of specific changes in diet quality or total energy intake (17). Unlike traditional dietary interventions, TRE does not necessarily impose explicit caloric targets or restrictions on food composition (17). Experimental and clinical investigations indicate that this eating pattern can improve several health-related outcomes, including body weight regulation, insulin sensitivity, glycemic control, reduced blood pressure, and improved lipid profile (18, 37–40).

Early clinical trials exploring this approach have primarily consisted of short-term behavioral interventions conducted in adults with overweight or obesity. Evidence describes moderate eating windows (6–10 h) as feasible, well tolerated, and associated with positive metabolic outcomes. Very short eating windows (e.g., ~4 h) do not appear to provide additional advantages and could be related to lower adherence or minor adverse effects, whereas longer windows (e.g., ~12 h) may yield limited or no significant benefits (17).

Paoli et al. (9) highlighted a shift in the traditional view that higher meal frequency is inherently beneficial, emphasizing emerging research linking frequent eating patterns to increased disease risk. On the other hand, more structured eating patterns characterized by reduced meal frequency and regular fasting intervals may confer metabolic advantages, including lower inflammation, improved circadian rhythmicity, modulation of the gut microbiota, and enhanced cellular processes, particularly autophagy and stress resistance.

Autophagy is a conserved cellular process activated in response to nutrient deprivation, as initially demonstrated in yeast models by the Nobel Prize winning Ohsumi et al. (41, 42). Translational data showed that fasting-related interventions in humans may promote metabolic switching and cellular stress responses, including the upregulation of autophagy-mediated pathways. These adaptations have been associated with improvements in insulin sensitivity, reduced oxidative stress and inflammation, and greater metabolic resilience, supporting the potential role of fasting-based strategies in promoting cardiometabolic health (19, 43).

Beyond autophagy, TRE may influence several interconnected nutrient sensing and circadian pathways involved in metabolic regulation. During fasting periods, reductions in nutrient availability and circulating insulin levels can activate AMP activated protein kinase (AMPK) and sirtuin 1 (SIRT1), while suppressing mechanistic target of rapamycin (mTOR) signaling. These adaptations favor lipolysis, decreased oxidative stress, and improved mitochondrial function (19, 43). Prolonged fasting intervals can also induce metabolic switching from glucose utilization toward increased fat oxidation and ketogenesis, which has been associated with metabolic flexibility and insulin sensitivity (43). Nutrient sensing pathways interact closely with the molecular circadian clock through the transcriptional regulators CLOCK and BMAL1, suggesting that feeding-fasting cycles might modulate peripheral circadian rhythms and downstream metabolic processes (44). These mechanisms provide a plausible biological framework through which TRE may influence metabolic health beyond caloric restriction alone.

## Evidence linking time-restricted eating to weight loss and metabolic health

Findings from experimental studies reveal that TRE may promote weight loss and improvements in metabolic health, even though results remain inconsistent across study designs and populations. Table 1 summarizes the main clinical and experimental studies evaluating TRE and metabolic outcomes.

**Table 1:** Summary of clinical and experimental evidence on time-restricted eating and metabolic health outcomes.

Study	Population	TRE protocol	Duration	Main findings
Sutton et al. (18)	Men with prediabetes	Early TRE (6 h eating window, last meal before 3 p.m.) or a 12-h feeding period	5 weeks	Improved insulin sensitivity, *β*-cell responsiveness, blood pressure, oxidative stress, and appetite without significant weight loss
Lowe et al. (59)	Adults with overweight or obesity	8-h TRE (12-8 p.m.) vs. consistent meal timing with 3 structured meals per day	12 weeks	No significant differences in weight loss or metabolic parameters between groups
Moro et al. (37)	Resistance-trained participants	8-h TRE combined with resistance training	12 months	Reduced body weight, fat mass, inflammatory markers and improved cardiometabolic risk factors
Phillips et al. (50)	Adults with at least 1 component of metabolic syndrome	12-h TRE vs. standard dietary advice	12 weeks	Significant weight loss from baseline, but no significant differences between groups
Pavlou et al. (60)	Adults with type 2 diabetes	8-h TRE vs. daily caloric restriction vs. control	6 months	Greater weight loss compared with controls, but no superior glycemic improvements versus caloric restriction
Steger et al. (61)	Adults with obesity	Early 8-h TRE plus caloric restriction	14 weeks	No improvements in diet quality or eating behavior compared with caloric restriction alone
Manoogian et al. (45)	Adults with metabolic syndrome	8-10-h TRE	3 months	Reductions in body weight, fat mass, and improved glycated hemoglobin
Bohlman et al. (56)	Adults from randomized controlled trials	Various TRE protocols (daily fasting duration ≥14 h)	At least 8 weeks (systematic review)	TRE generally did not worsen sleep outcomes
Clavero-Jimeno et al.(55)	Adults with overweight or obesity	Early, late, or self-selected 8-h TRE	12 weeks	No significant effects on sleep, mood, or quality of life
Chen et al. (39)	Adults from randomized trials	Early vs. delayed TRE	Network meta-analysis	Early TRE showed greater improvements in anthropometric and glycemic outcomes
Kramer et al. (57)	Adults with overweight/obesity and type 2 diabetes	4-h TRE	6 weeks per intervention plus 4-week washout	TRE induced weight loss, but did not prevent compensatory hormonal adaptations associated with weight reduction

Restricting the daily eating window to approximately 8–10 h has been linked to beneficial changes in glycemic regulation and reductions in body weight, BMI, and body fat mass in individuals with metabolic syndrome. Most of the weight loss appears to be attributable to reductions in fat mass, with minimal loss of lean mass, indicating a favorable body composition profile. Part of these effects could be explained by a spontaneous reduction in caloric intake, but the structured eating window itself may contribute to physiological regulation by consolidating feeding-fasting cycles and promoting better alignment with circadian physiology (45). Thus, TRE can confer benefits across a range of cardiometabolic diseases, including obesity, T2DM, cancer, fatty liver disease, and cardiovascular disease (46, 47). In addition to weight-related outcomes, some trials have reported benefits in other cardiometabolic markers. Early TRE protocols have been associated with enhancements in insulin sensitivity and lipid profiles, as well as reductions in inflammatory markers, blood pressure, and oxidative stress (37, 38), even in the absence of significant weight loss (18).

The duration and timing of the eating window are important factors influencing the effects of TRE. Interventions using moderate eating windows (e.g., 10 h) have been connected to greater weight loss compared with longer windows (e.g., 12 h), likely reflecting differences in fasting duration and potential effects on energy intake (48). Longer windows (e.g., 12 h) tend to show limited or no significant additional health benefits compared with more moderate TRE protocols (17, 49, 50). These discoveries suggest that a moderate restriction of the eating window may represent a more effective and sustainable approach for improving health-related outcomes.

A systematic review and meta-analysis found that early TRE combined with caloric restriction resulted in greater reductions in body weight and fat mass compared with delayed TRE (51). In another meta-analysis, women with overweight and obesity experienced reductions in body weight and fasting insulin levels with TRE compared to conventional diets, but not when compared to caloric restriction alone. Lean mass was preserved, emphasizing the safety of TRE strategies in weight loss (52). Chang et al. (53) concluded that the health benefits of TRE are primarily driven by energy deficit, with circadian alignment acting as a secondary contributing factor.

A recently published systematic review and network meta-analysis observed that overall TRE, compared to usual diets, improved body weight, BMI, body fat mass, waist circumference, systolic blood pressure, and fasting blood glucose, insulin, and triglycerides. Early TRE was more beneficial for anthropometric measurements, body weight, glycemic parameters, and fasting insulin concentrations compared to usual diets and delayed TRE (39). Liu et al. (40) indicated reductions in body weight and fat mass, and improvements in lipid parameters.

Sleep can also be influenced by TRE. In a discussion by Benedict and Heilbronn, protocols involving eating windows of approximately 9–10 h were connected with better subjective sleep quality. The authors noted that evidence for objective sleep outcomes is still inconsistent, with some studies indicating no significant changes or even potential impairments. These discrepancies might be related to factors, such as increased hunger or psychological stress associated with adherence, highlighting the need for further investigation into the effects of TRE on sleep architecture and physiology (54). A randomized controlled trial did not find significant differences in objective sleep parameters between the TRE and the control group (55). Bohlman et al. (56) systematic review pointed out that short- to mid-term TRE did not appear to worsen sleep parameters, but responses may vary across individuals, pointing to the need for further research.

The current body of experimental data proposes that TRE can induce small but consistent changes in body weight and selected metabolic outcomes. The magnitude and consistency of these effects depend on multiple determinants, including intervention duration, baseline metabolic status, energy intake, and the timing of the eating window. These inconsistencies emphasize the need for careful interpretation of findings and underscore the importance of methodological considerations, which are discussed in the following section.

## Current controversies and methodological challenges

Although TRE can promote spontaneous caloric restriction and modest weight loss, recent evidence identified that it did not attenuate the compensatory hormonal adaptations to weight reduction, for example decreases in leptin and increases in ghrelin, which can favor weight regain. It is important to consider the characteristics of the intervention protocol, as this particular study employed a relatively short eating window (4 h) (57), which may influence both adherence and physiological responses. Extreme fasting protocols might negatively influence adherence and tolerability without consistently improving metabolic outcomes (17, 49, 50).

Some randomized controlled trials have revealed null or inconclusive results (55, 58, 59). For example, the TREAT randomized clinical trial described no significant differences in weight loss and metabolic parameters between participants assigned to an 8-h eating window and those following a standard eating schedule (59). Clavero-Jimeno et al. (55) documented no significant changes in sleep, mood, or quality of life in adults with overweight or obesity undergoing different 8-h TRE protocols (early, late, or self-selected) compared with a usual care group receiving dietary counseling after 12 weeks. Similarly, Pavlou et al. (60) demonstrated that while TRE resulted in greater weight loss compared with control conditions, it did not lead to superior improvements in glycemic control when compared with daily caloric restriction, suggesting comparable metabolic effects between interventions.

In their review, Lages et al. (38) identified substantial heterogeneity across TRE studies, particularly regarding metabolic, inflammatory, and circadian outcomes. Inflammatory markers, particularly C-reactive protein (CRP) and tumor necrosis factor alpha (TNF-*α*), showed inconsistent results, with few analyses finding statistically significant changes, and lipid profile responses were variable and often not significantly different from control groups. Even though reductions in glucose levels were frequently observed, these changes were not consistently significant when compared to control conditions in most reports (38).

The same authors highlighted the potential role of circadian regulation in mediating TRE effects. TRE interventions were associated with alterations in clock gene expression and genes involved in metabolic regulation in adipose tissue, supporting an interaction between feeding-fasting cycles and peripheral circadian clocks (38). Data on circadian, inflammatory, and oxidative stress biomarkers remains limited and occasionally contradictory, reinforcing the need for further well-controlled research. Consistent with these findings, Steger et al. (61) reported that early TRE combined with caloric restriction did not improve diet quality, eating behavior, or meal frequency compared with caloric restriction alone, and could lead to greater hunger during fasting periods.

Experiments combining TRE with caloric restriction have not consistently indicated additional metabolic benefits beyond those achieved through caloric restriction alone. In their systematic review and meta-analysis, Sun et al. (51) stated no significant effects on cardiometabolic markers, including blood pressure, glucose profile, and lipid profile, in TRE combined with caloric restriction when compared to caloric restriction alone. In women with overweight and obesity, TRE had no significant effects on BMI, body fat mass, visceral fat, lipid and glucose profile or blood pressure (52). TRE presented improvements in body weight, fat mass, and lipid markers in individuals with overweight; however, it had no effects on waist circumference, BMI, glycosylated hemoglobin, or blood pressure (40). Interpretation of these outcomes is further complicated by differences in TRE protocols, intervention length, participant groups, and inclusion and exclusion criteria.

## Research gaps and future directions

Despite the growing body of evidence, important gaps remain in the current literature on TRE. A major limitation is the predominance of short-term approaches (17, 49, 58), which restricts the understanding of long-term sustainability, effectiveness, and potential compensatory behaviors related to weight management and metabolic health.

Heterogeneity across study designs represents another key challenge. Differences in eating window duration, timing (early versus delayed TRE), intervention length, and population characteristics limit comparability and hinder the establishment of consistent recommendations (17, 38, 39, 58). Standardized, well-controlled randomized trials are essential to improve consistency and interpretability across findings.

Further investigation of protocols adapted to diverse population profiles is warranted. This includes individuals with distinct chronotypes, shift workers, and those with sleep disorders. Direct comparisons between early and late eating windows, under tightly controlled energy intake and diet quality conditions, are particularly relevant for clarifying underlying mechanisms. Integrating clinical and behavioral outcomes, such as adherence, hunger perception, sleep quality, and psychological wellbeing, would provide a more comprehensive evaluation of TRE under real-world conditions.

The role of circadian biology is still insufficiently characterized. Most TRE approaches do not account for individual chronotype, despite its potential influence on metabolic responses to meal timing (8, 15, 16). Aligning eating schedules with endogenous circadian preferences could improve metabolic efficiency, especially in individuals with evening chronotypes, who tend to present higher cardiometabolic risk (15, 35, 62).

Uncertainty persists regarding the relative contribution of caloric restriction and circadian alignment. Although TRE frequently induces spontaneous caloric reduction, it remains unclear whether observed benefits primarily reflect reduced energy intake or improved temporal synchronization, with current data suggesting a dominant role of energy deficit (51, 53). Experimental designs incorporating strict control of energy intake are critical to disentangle these effects. This strategy would help determine whether TRE exerts independent metabolic actions, supporting more precise and mechanism-based clinical recommendations.

Findings on non-metabolic outcomes continue to be limited and inconsistent (54–56). Expanding the scope of investigation to include sleep, mental health, and quality of life may clarify whether TRE influences broader physiological and behavioral domains. This line of inquiry could determine if TRE functions as an integrative intervention capable of modulating multiple systems simultaneously.

Advancing these research directions can cooperate with the development of more refined and applicable nutritional strategies. The inclusion of circadian variables, particularly chronotype, through simple and low-cost validated questionnaires, including the Morningness-Eveningness Questionnaire (MEQ) and the Munich Chronotype Questionnaire (MCTQ), offers a practical opportunity to personalize meal timing interventions and optimize dietary guidance. These tools may help identify individual circadian preferences and guide the scheduling of eating windows according to biological and behavioral patterns. Individuals with earlier chronotypes may better tolerate earlier eating windows, whereas later chronotypes could benefit from progressively adjusted meal timing strategies designed to reduce circadian misalignment. Over time, chrononutrition-based approaches have the potential to improve motivation and adherence, enhance wellbeing, and promote greater autonomy in health management. At the population level, these strategies may eventually contribute to more accessible and individualized dietary guidance, although their clinical and public health impact still requires further validation in well-controlled long-term clinical trials before widespread implementation in clinical practice.

## Discussion

TRE has emerged as a relevant area within chrononutrition, attracting attention as a non-invasive, behavior-based dietary strategy (8, 17, 19). Current evidence documents modest reductions in body weight and improvements in selected metabolic parameters, particularly when compared to habitual eating patterns (39, 40, 45).

When contrasted with conventional hypocaloric diets, results are less consistent. Accumulated data support the notion that the metabolic effects of TRE are strongly influenced by reductions in energy intake (51, 53, 60). Improvements noted within TRE groups relative to baseline imply that structured eating windows can still be an effective behavioral strategy (39, 45).

Long-term sustainability remains an important consideration. While short- to mid-term interventions indicate that TRE is generally viable and well tolerated, adherence over an extended period is uncertain and likely influenced by behavioral and contextual factors, including hunger, social constraints, and lifestyle compatibility (17, 49, 61).

Circadian rhythms are often under recognized in clinical practice, despite their relevance for metabolic regulation (12, 30). Greater dissemination of chrononutrition concepts among healthcare professionals could favor the implementation of dietary strategies that incorporate not only dietary composition and quantity, but also timing of food intake (8, 10). Training in circadian medicine is feasible and may yield relevant benefits in clinical practice by promoting a more integrated approach to eating behavior, increasing intervention effectiveness, and encouraging sustained adherence over time.

Most TRE protocols overlook chronotype, which can result in suboptimal alignment between feeding schedules and endogenous rhythms. Incorporating this variable into dietary planning could enhance metabolic responses and represent a promising direction for tailored strategies (15, 35). Aligning recommendations with biological preferences and daily routines might also improve motivation, perceived effectiveness, and overall satisfaction, key factors for long-term behavioral change.

Insomnia, a highly prevalent sleep disorder (63), is a relevant, yet underexplored, target for TRE interventions. Individuals with insomnia are at greater risk of overweight and obesity, both indirectly, through metabolic dysregulation associated with sleep deprivation (64), and directly, due to prolonged wakefulness that increases opportunities for food intake (65, 66), particularly during the biological night, when metabolic efficiency is reduced (23, 25). The expansion of daily eating windows commonly observed in this population suggests that TRE could help limit nocturnal intake and improve cardiometabolic outcomes.

Clinical trials focusing on this group could evaluate body weight, body composition, nighttime eating patterns, and metabolic markers. Restricting food intake during the biological night may provide a key advantage, with potential benefits for both weight regulation and metabolic health in this vulnerable population.

TRE characterizes a flexible and viable dietary approach, particularly in comparison with unrestricted eating patterns (39, 45). Its effectiveness relative to caloric restriction remains uncertain and appears to depend on multiple interacting factors, including adherence, energy intake, and circadian alignment (51, 53). Well-designed, long-term randomized controlled trials examining the interaction between meal timing, caloric intake, and individual circadian profiles are essential to define the contexts in which TRE is most effective.

Clarifying these relationships would expand the clinical applicability of TRE and support the development of more precise and targeted recommendations. In a broader context, this knowledge may contribute to scalable, low-cost, behavior-based strategies with meaningful impact on the prevention and management of chronic diseases.
